# Evaluation of *APOBEC3* expression as prognostic marker in squamous cell carcinoma of the penis

**DOI:** 10.1038/s41598-022-17056-8

**Published:** 2022-07-28

**Authors:** Bettina Trimmel, Andre Oszwald, Christoph Diemand, Iris E. Ertl, Ursula Lemberger, Andreas Bruchbacher, Robert Brettner, Stephan Korn, Irene Resch, Eva Comperat, Shahrokh F. Shariat, Melanie R. Hassler

**Affiliations:** 1 Department of Pathology, Medical University of Vienna, Vienna, Austria; 2 Department of Urology, Comprehensive Cancer Center, Vienna General Hospital, Medical University of Vienna, Währinger Gürtel 18-20, 1090 Vienna, Austria; 3 Hourani Center for Applied Scientific Research, Al-Ahliyya Amman University, Amman, Jordan; 4 Department of Urology, The University of Texas Southwestern Medical Center, Dallas, TX USA; 5 Department of Urology, Weill Cornell Medical College, New York, NY USA; 6 Department of Urology, Second Faculty of Medicine, Charles University, Prague, Czech Republic; 7 Institute for Urology and Reproductive Health, I.M. Sechenov First Moscow State Medical University, Moscow, Russia

**Keywords:** Penile cancer, Viral infection, Tumour biomarkers, Gene expression

## Abstract

Squamous cell carcinoma of the penis (PSC) is a rare disease with limited information on the molecular events leading to malignant transformation. In a third of PSC cases, presence of human papilloma virus (HPV) is found. The APOBEC3 family of proteins is known to play a significant role in defense against HPV infection, but their role in PSC is largely unknown. In this study, we aim to assess mRNA expression levels of APOBEC3 family members in HPV+ and HPV− PSC to get insight into their association with clinicopathological features and to evaluate their prognostic impact. Expression levels of six *APOBEC3* family members in tissue from 50 patients with PSC were determined by RT-PCR and correlated with clinical and histopathological features. Lower expression of *APOBEC3A*, *APOBEC3B,* and *APOBEC3C* was observed in advanced PSC stages. Except for *APOBEC3D*, HPV+ samples showed higher expression of *APOBEC3s* compared to HPV− samples. In univariate analyses, *APOBEC3A* and *APOBEC3C* expression tended to be associated with disease-free survival and *APOBEC3A* expression with overall survival; however, multivariable analyses failed to confirm these associations with outcome. More extensive external validation and functional laboratory studies are needed to evaluate further their role in PSC development and progression.

## Introduction

Squamous cell carcinoma of the penis (PSC) is a rare disease with an incidence of 1.6 per 100.000 men^1^. Risk factors comprise phimosis, chronic penile inflammation, smoking, low-socioeconomic status, and infection with human papillomavirus (HPV)^2^. Approximately a third of PSC cases is associated with HPV infection^3^.

Human cells infected by viruses can utilize several anti-viral defense strategies to inhibit viral replication, among which members of the Apolipoprotein B mRNA Editing Catalytic Polypeptide-like 3 (APOBEC3) family of proteins play a significant role^4^. The APOBEC3 family comprises seven genes (*APOBEC3A*, *APOBEC3B*, *APOBEC3C*, *APOBEC3D*, *APOBEC3F*, *APOBEC3G,* and *APOBEC3H*) located within a cluster on chromosome 22.q13.1^5^. Due to their ability to de-aminate cytidine to uridine in ssDNA, they generate mutations in viral genomes after infection of cells with different viruses such as HIV or HPV^6,7^. Besides their function in innate anti-viral immunity, an APOBEC-induced mutational signature has been detected in the genomes of several cancers^8^. The occurrence of this mutational signature may be due to increased APOBEC3 activity, which leads to cellular DNA damage.

It is currently not clear which APOBEC3 enzymes are induced in HPV+ cells and serve as defense against viral in fection and which are responsible for APOBEC mutational signatures in the cancer genome. In PSC, APOBEC mutational signatures have been reported to be enriched in tumors with a high viral load, but only APOBEC3A protein expression has been studied in PSC tumors^9,10^. Protein expression of APOBEC3A was reduced in invasive stages of HPV-negative PSC, whereas APOBEC3A expression was maintained in HPV+ tumors. To date, we are not aware of studies reporting data on the expression of further APOBEC3 family members in PSC.

In order to investigate *APOBEC3* gene family expression in PSC, we determined mRNA expression levels of six APOBEC3 family members in tumor tissue from 50 PSC patients and correlated individual expression levels with clinicopathological features and survival outcomes.

## Results

### Association of *APOBEC3* expression with clinical and histopathological parameters in penile squamous cell carcinoma

**Figure 1:**
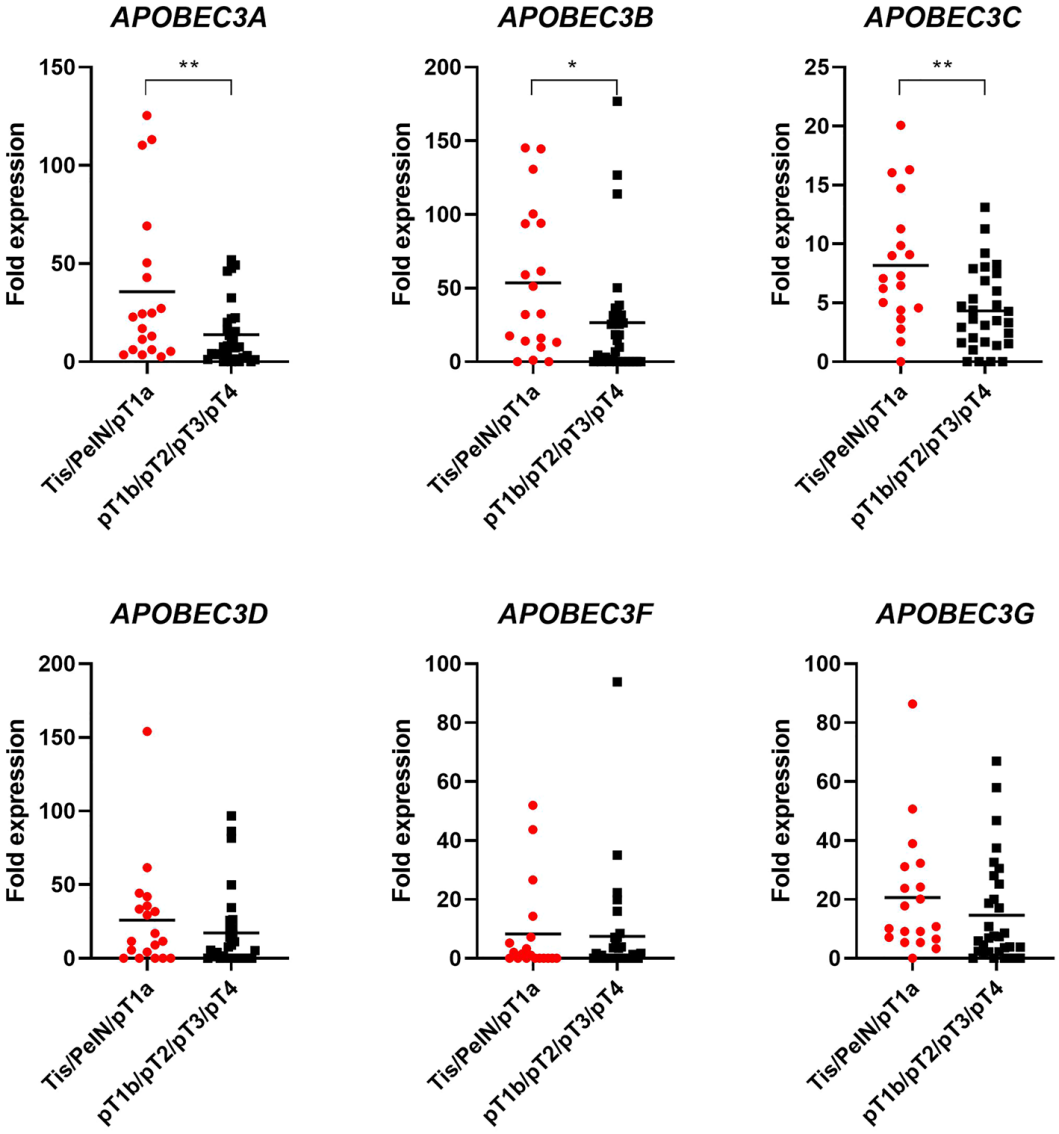
*APOBEC3* mRNA expression in tumor tissue from penile squamous cell carcinoma patients decreases in advanced stages. mRNA expression levels for individual *APOBEC3s* for samples < pT1b (n = 19) and ≥ pT1b (n = 31) are shown. * = *p* < 0.05, ** = *p* < 0.001.

**Figure 2:**
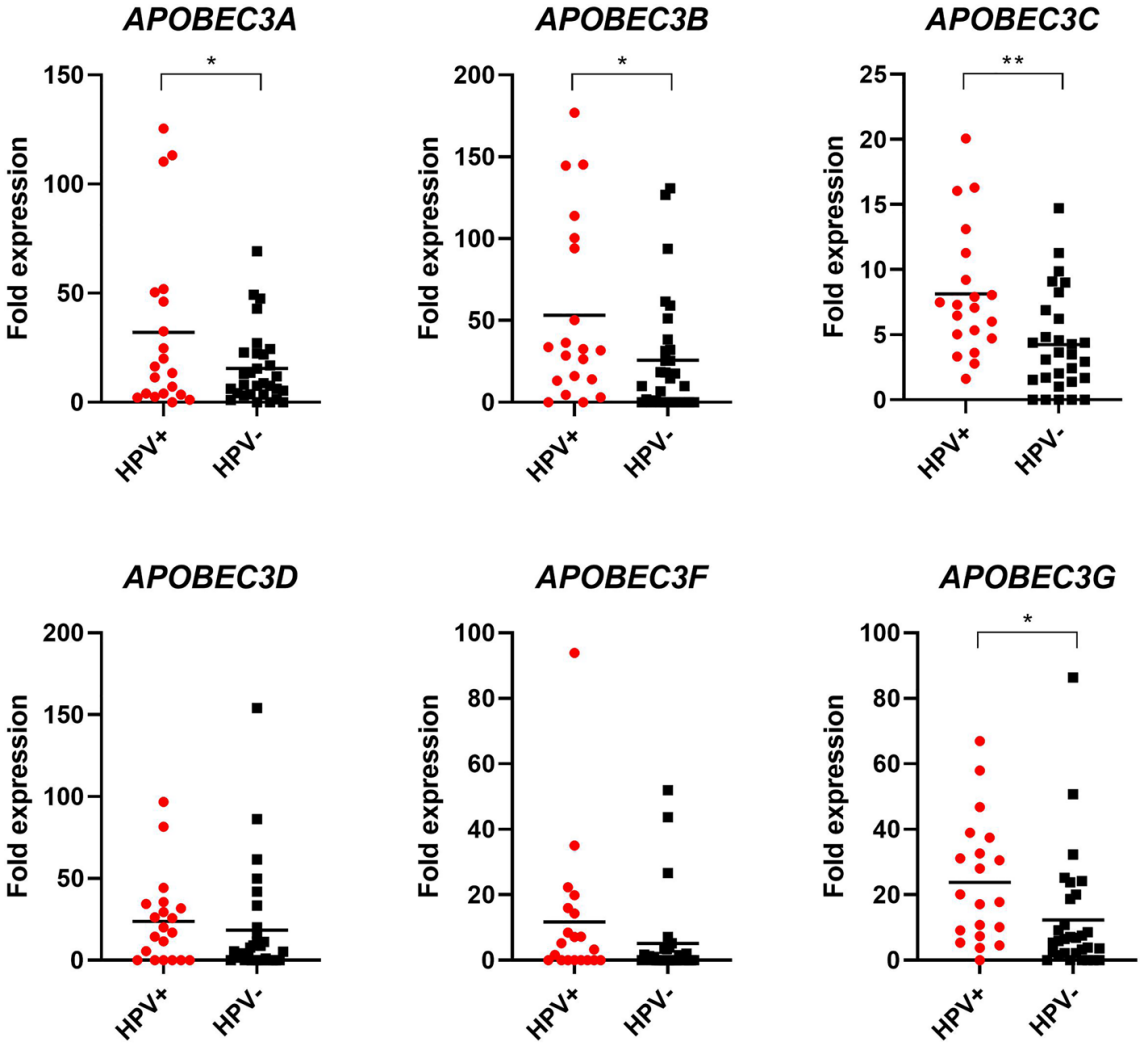
Association of *APOBEC3* mRNA expression with HPV status. mRNA levels for individual *APOBEC3s* in relation to HPV status are shown. n_HPV+_  = 20, n_hpv−_ = 30. * = *p* < 0.05, ** = *p* < 0.001.

**Figure 3:**
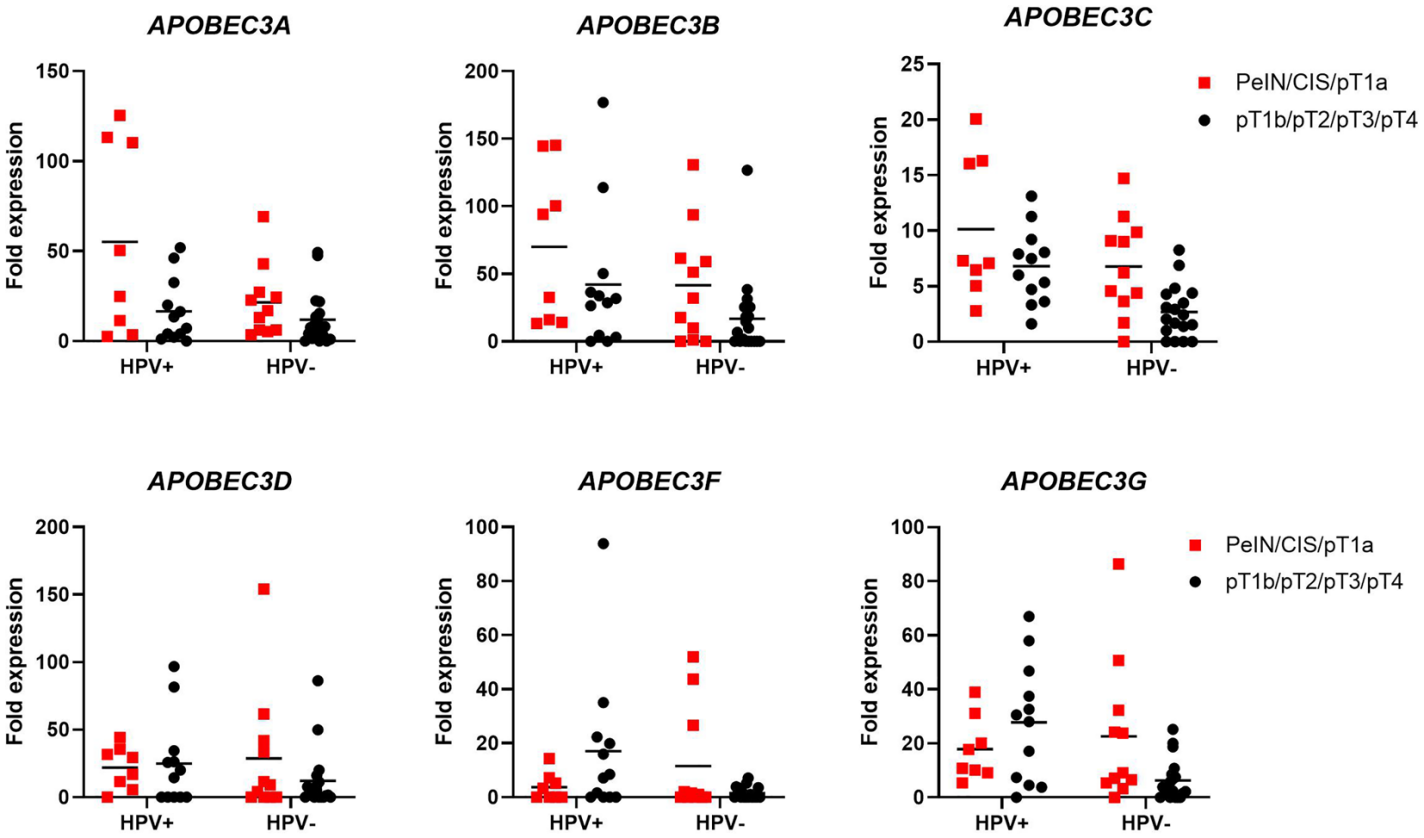
Association of *APOBEC3* mRNA expression with HPV status and stage. mRNA levels for individual *APOBEC3s* in relation to HPV status and stage are shown. n_HPV+/<pT1b_ = 8, n_HPV+/>pT1b_ = 12, n_HPV−/<pT1b_ = 11, n_HPV−/>pT1b_ = 19. * = *p* < 0.05, ** = *p* < 0.001.

In total, 50 patients with PSC were included in this study. Patient characteristics are shown in Table 1. The cohort's median age was 64 years (IQR 52–73). pT1b stage and higher was present in 31 patients (62%), 18 patients (36%) underwent inguinal lymphadenectomy. The median follow-up time was 24 months (IQR 1–62). We first evaluated the association of individual *APOBEC3* gene expression with clinical parameters (age, stage, grade, histological subtype, lymph node, and HPV status) in our cohort. No significant correlation with age or grade was observed (Supplementary Table 2). Regarding stage, *APOBEC3* expression tended to be lower in tumors of advanced stages (> pT1a), with a significant difference observed for *APOBEC3A*, *APOBEC3B,* and *APOBEC3C (*Fig. 1*)*. After stratification into low- and high expressing samples, this difference remained significant for *APOBEC3C* (Supplementary Table 2). A trend towards a significant difference between low- and high expressing samples was also seen regarding histological subtype for *APOBEC3A* and lymph node status for *APOBEC3C* (Supplementary Table 2). Except for *APOBEC3D*, *APOBEC3* expression was increased in HPV+ compared to HPV− samples (Fig. 2). A significant difference was seen for *APOBEC3B* and *APOBEC3C*, and, after stratification, *APOBEC3G*. Additionally, among HPV+ samples, *APOBEC3A* was significantly down-regulated in ≥ pT1b PSCs compared to tumors of lower stages, whereas this effect was not as pronounced among HPV− samples. Also, HPV+  < pT1b samples had significantly higher *APOBEC3A* expression than HPV−  < pT1b samples. Although showing a similar trend, *APOBEC3B* and *APOBEC3C* expression levels were not significantly different between HPV+ and HPV− cases. No differences were observed between *APOBEC3D*, *APOBEC3F,* or *APOBEC3G* expression and HPV status concerning tumor stage (Fig. 3).

**Table 1:** Clinical characteristics of penile squamous cell carcinoma patients.

Clinical characteristics	*n* = 50
**Age**	
Median (range)	64 (31–90)
**Type surgery (%)**	
Biopsy	1 (2)
Local excision/circumcision	11 (24)
Glansectomy	13 (26)
Partial penectomy	17 (34)
Total penectomy	8 (16)
**pT (%)**	
PeIN	1 (2)
CIS	3 (6)
pT1a	15 (30)
pT1b	3 (6)
pT2	21 (42)
pT3	6 (12)
pT4	1 (2)
**Grade (%)**	
G1	7 (14)
G2	26 (52)
G3	12 (24)
NA	5 (10)
**cN (%)**	
cN0	12 (24)
cN+	9 (18)
cNx	29 (58)
**pN (%)**	
pN0	5 (10)
pN+	13 (26)
pNx	32 (64)
**Subtype (%)**	
Usual type	25 (50)
Warty/basaloid/warty-basaloid	8 (16)
Sarcomatoid	4 (8)
Pseudohyperplastic	4 (8)
Other	9 (18)
**inguinal LAE (%)**	
Yes	18 (36)
No	32 (64)
**HPV status (%)**	
Positive	20 (40)
Negative	30 (60)

*NA* not reported.

### Evaluation of *APOBEC3* expression as a prognostic marker

**Figure 4:**
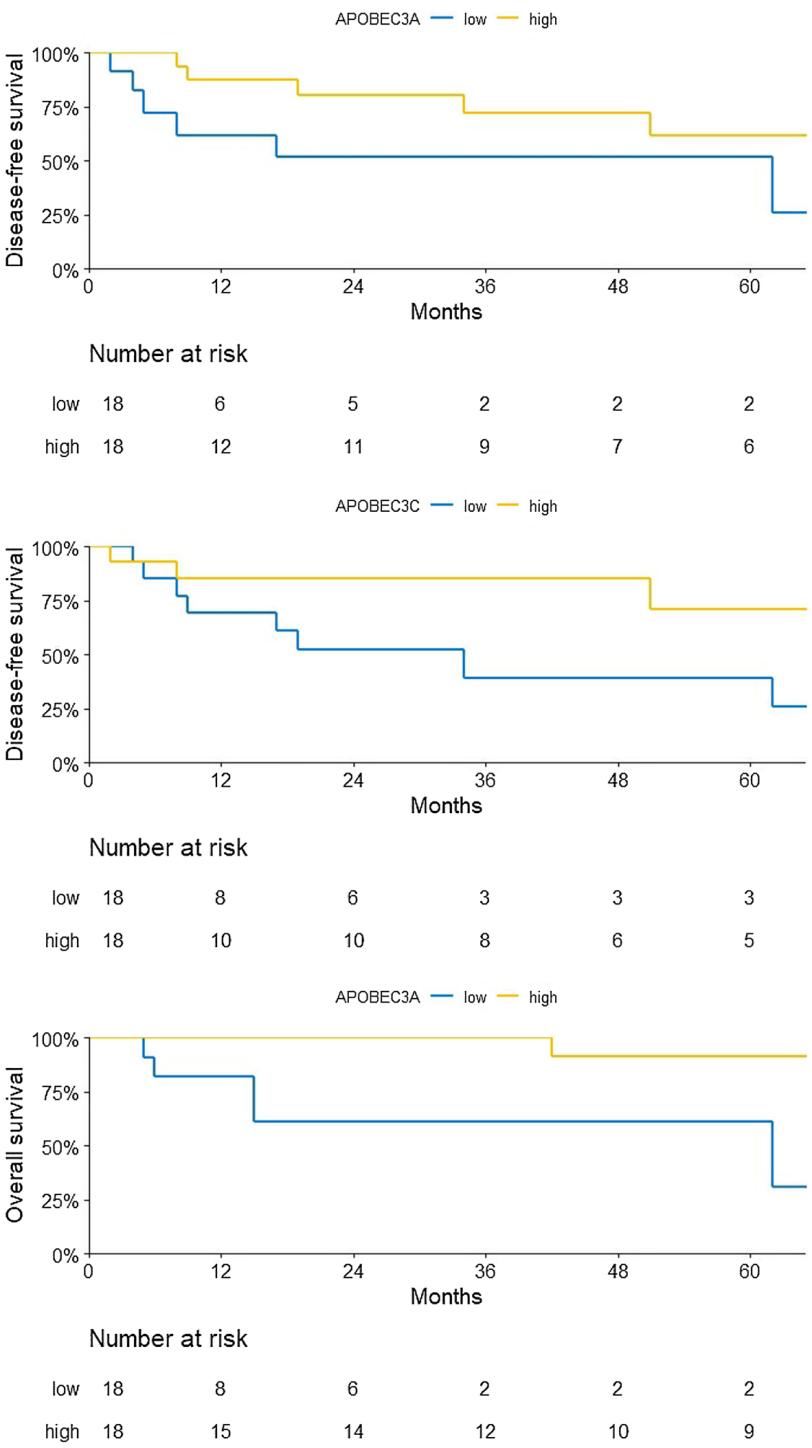
Association with *APOBEC3* mRNA expression and DFS or OS. A trend towards an association is detected for expression of *APOBEC3A* and *APOBEC3C* with DFS and a significant association for expression of *APOBEC3A* with OS. n_total_ = 36, n_*APOBEC3A/C* mRNAhigh_ = 18, n_*APOBEC3A/C* mRNAlow_ = 18.

We next investigated individual *APOBEC3* expression as a prognostic marker in PSC patients with < pT1b or ≥ pT1b and inguinal lymphadenectomy (LAE) (n = 36), as we reasoned that patients without adequate LAE would have worse survival and thus should not be included in prognostic marker analysis. On univariate analyses, there was a trend towards an association of *APOBEC3A* and *APOBEC3C* mRNA^high^ with disease-free survival (DFS) (HR 0.35, CI 0.10–1.15, *p* = 0.08; HR 0.29, CI 0.08–1.12, *p* = 0.07) and a significant association of *APOBEC3A* mRNA^high^ with overall survival (OS) (HR 0.14, CI 0.03–0.76, *p* = 0.02) (Fig. 4). On multivariable analyses adjusting for the effects of pathologic stage and lymph node status, *APOBEC3A* mRNA^high^ did not remain significantly associated with OS. None of the other *APOBEC3s* was associated with neither DFS nor OS (Supplementary Table 3).

## Discussion

In this study, we investigated the association of mRNA expression levels of six *APOBEC3* genes with clinical features of PSC and found that expression of *APOBEC3A*, *APOBEC3B,* and *APOBEC3C* decreases in higher PSC stages, indicating that *APOBEC3* expression seems to be down-regulated in advanced PSC. Furthermore, except for *APOBEC3D*, higher *APOBEC3* expression was seen in HPV+ samples, which is in line with the finding that APOBEC3A, APOBEC3B, APOBEC3C, APOBEC3F, and APOBEC3G restrict DNA viruses such as HPV^7,11^. Up-regulation was especially prominent for *APOBEC3A* in low-stage HPV+ PSCs compared to advanced HPV+ PSCs. This up-regulation is likely due to cellular response after HPV infection in PSCs. In the early stages of HPV infection, APOBECs act as restriction factors by inducing mutations in HPV genomes^7,11^. For cervical cancer, whole-genome HPV16 sequencing has shown that a high load of viral APOBEC3 mutations due to high APOBEC3 activity is associated with benign or clearing HPV16 infections^12^. APOBEC3A seems to be a more potent restriction factor for HPV infectivity than the other APOBEC3s^13^. In our study, HPV+ samples with high *APOBEC3A* expression had a better outcome than HPV+ samples with low *APOBEC3A* expression, which was observed for both low-stage and advanced (≥ pT1b) PSC samples. Based on this and previous observations, we hypothesize that increased *APOBEC3A* expression may be associated with clearing of HPV infections and low-risk disease. In case of reduced APOBEC3s expression, HPV-induced oncogenic driver events may contribute to a larger extend to malignant transformation.

APOBEC enzymes also cause APOBEC-specific mutations in the tumor genome and contribute to genomic instability. In PSC, an APOBEC signature has been reported to be present in up to 38% of cases. The presence of this signature was associated with the presence of lymph node metastasis and worse OS. Furthermore, the APOBEC signature was positively correlated with tumor mutational burden^14^. Data from other cancers have shown that APOBEC3B is the primary enzyme responsible for APOBEC signature-related mutations^15,16^, whereas APOBEC3A seems to be inducing DNA damage via double-strand breaks to a higher extend than APOBEC3B^17^. APOBEC3A and APOBEC3B often function as oncogenes in these cancers. In contrast, our study found an association between *APOBEC3A* expression and longer OS, which would assign tumor-suppressive functions to APOBEC3A in PSC. Indeed, a study evaluating APOBEC3A protein expression in PSC found reduced APOBEC3A protein expression in the invasive parts of HPV-negative PSCs^9^. In our study, higher *APOBEC3A* expression was found in HPV+ low-stage cases compared to HPV+ advanced stages and HPV− cases. For the other APOBEC3s, no such correlation was observed. This leads us to speculate that *APOBEC3A*—having tumor-suppressive functions in HPV+ disease—is not responsible for APOBEC-induced mutational signatures correlated with worse outcomes in HPV+ PSCs. Finally, although higher *APOBEC3A* expression was significantly correlated with longer OS in univariate analysis, no correlation of *APOBEC3A* expression and OS was found in multivariable analysis when stage was added, highlighting the interplay of *APOBEC3A* expression and stage.

Our study is affected by several limitations. From the clinical point of view, not all patients in our cohort received inguinal lymph node dissection, which is the current standard of treatment for PSC patients with ≥ pT1b. Furthermore, we could not interrogate up- or down-regulation of APOBEC3s on the protein level, as commercially available antibodies lack specificity for individual APOBEC3s. We also did not correlate APOBEC3 overexpression with APOBEC signatures. Finally, we report on a relatively small sample size, which may not be adequately powered for analyzing modest differences between APOBEC3 expression and correlation with clinical outcomes.

Still, our study is the first to report on expression levels of six *APOBEC3s* in PSC, extending previous data on APOBEC3A protein expression only. We can confirm a positive correlation between low-stage PSCs, HPV infection, and *APOBEC3* expression. Furthermore, we show that *APOBEC3A* expression is especially pronounced in low-stage HPV+ cases and is correlated with OS in PSC. In conclusion, expression of *APOBEC3* genes in PSC is associated with less aggressive disease stages and the presence of HPV. Future studies involving larger patient cohorts are necessary to validate their prognostic value and their specific roles in histological types across the disease spectrum.

## Materials and methods

### Patient cohort

We obtained formalin-fixed, paraffin-embedded (FFPE) tissue samples from 50 consecutive archived PSC cases at the Medical University of Vienna. Patients were identified retrospectively and had received treatment from 1993 to 2019 (Table 1). FFPE tumor samples were histopathologically checked to confirm the presence of significant tumor tissue per sample and a dedicated uropathologist re-analyzed tumor samples according to the 2016 UICC TNM classification. Specimens from FFPE material for RNA isolation were obtained after approval by the institutional ethics committee of the Medical University of Vienna (EK 1456/2019).

### Primer design, RNA extraction, and qRT-PCR

Primer design for APOBEC3A-G was performed by Primer3 software (https://primer3.ut.ee) after obtaining gene sequences from the UCSC Genome Browser (GRCh38g/hg38) (https://genome.ucsc.edu). Primer pairs were selected so that paired nucleotide sequences remained specific for amplification of individual *APOBEC3s* and did not overlap with homologous regions of non-target genes, thus eliminating off-target amplification. *ACTB* was used as reference gene. Primer pairs are shown in Supplementary Table 1.

For gene expression analysis, RNA was extracted from FFPE tissues using the RNAeasy FFPE kit (Qiagen) according to protocol. RNA concentration was measured using the DeNovix fluorescence assay system (DeNovix). For qRT-PCR quantification, at least 250 ng of RNA were used as a template for random hexamer cDNA synthesis using TaqMan Reverse Transcription reagents (ThermoFisher Scientific) according to supplier’s protocol. qRT-PCR was performed with SybrGreen MasterMix (ThermoFisher Scientific) on the QuantStudio7 system (Applied Biosystems). 2 µl of cDNA was used per sample. Melting curve analysis and visualization of PCR products by gel electrophoresis were carried out to verify the presence of specific amplification products.

### HPV genotyping and p16 immunohistochemistry

For p16 immunohistochemistry (IHC), staining was performed with p16 E6H4 (Roche) according to the manufacturer’s protocol on a BenchMark ULTRA IHC/ISH Staining Module. The slides were deparaffinated at 72 °C with EZ-Prep (Ventana). Slides were then pretreated with Cell Conditioner 1 (Ventana) followed by Cell Conditioner 1 ULTRA CC1 protocol. Slides were subsequently incubated with p16 antibody for 20 min. Staining was performed with DAB Kit (Ventana). Counterstaining was performed with Hematoxylin (Ventana) and Bluing Reagent (Ventana).

HPV in situ hybridization (ISH) was performed using INFORM HPV II Family 6 (Roche, 800–2220) for HPV low risk, and INFORM HPV III Family 16 (Roche, 800-4295) for HPV high-risk strains. Staining of HPV ISH was carried out with Iview Blue Plus Detection Kit (Roche, 760-097) according to protocol. HPV III Family 16 detects HPV high risk strains 16, 18, 31, 33, 35, 45, 52, 56, 68 and 66. HPV II Family 6 detects low-risk strains and does not hybridize high-risk strains. Red Counterstain II (Ventana) was used for counterstaining.

A trained uropathologist evaluated the slides.

### Statistical analyses

Statistics for categorical variables included frequencies and proportions. The mean, median and interquartile range (IQR) were reported for continuous variables. The Mann–Whitney U test and the chi2-test were used to compare the statistical significance of differences in medians and proportions.

Survival analysis was performed using data obtained from the clinical follow-up. Overall survival was defined as months from surgery until patient death. Disease-free survival was defined as months until disease recurrence or progression. The association of *APOBEC3* expression with OS and DFS was assessed with the Kaplan–Meier method. Survival was compared between patients using the log-rank test.

P-values and hazard ratios were calculated by univariate and multivariable Cox regression models, including age, tumor stage, lymph node status, HPV status, and histological subtype. Statistical significance was considered at *p* < 0.1. All tests were two-sided.

For statistical analysis, R version 4.0.4 was used. Graphs were created with R or GraphPad Prism 8.

### Human participants and informed consent

This research study was conducted retrospectively and approved by the institutional ethics committee (ICB) of the Medical University of Vienna (EK 1456/2019). We consulted extensively with the ICB who determined that our study did not need informed consent, as information is anonymized and the submission does not include images that may identify the person.

## Supplementary Information

Supplementary Information 1.

Supplementary Information 2.

Supplementary Information 3.

Supplementary Information 4.

## Data Availability

All data generated or analyzed during this study are included in this published article.
