# Supplementary material for: Evaluation of APOBEC3 expression as prognostic marker in squamous cell carcinoma of the penis
Source: Sci Rep. 2022 Jul 28;12:12911. doi: 10.1038/s41598-022-17056-8 (PMC9334367; doi:10.1038/s41598-022-17056-8)
Supplement: Supplementary file 1 — Supplementary Information 1. [file 41598_2022_17056_MOESM1_ESM.docx]

**Supplementary Table 1** Primer sequences for *APOBEC3* mRNA expression analysis.

**Supplementary Table 2** Association of *APOBEC3* expression with clinical and histopathological parameters in PSC patients. n: number of patients; NA: not reported; SD: standard deviation

**Supplementary Table 3** Univariate and multivariable analyses assessing the association between predictor variables and DFS/OS among patients with PSC. CI: confidence interval; DFS: disease-free survival; HR: hazard ratio; OS: overall survival
